# New York Letter

**Published:** 1880-03

**Authors:** 


ARTICLE VIII.

### NEW YORK LETTER.

*Editors* CHICAGO MEDICAL JOURNAL AND EXAMINER:

The winter courses at the medical colleges are coming to an end, and with them the most important of the clinics stop. Chief among these clinics is that conducted by Professor James R. Wood, M.D., LL.D., in the amphitheater at Bellevue Hospital. "Jimmy Wood's Saturday clinics," are as familiar to the students and the profession here as Flint's Practice. They form a historical feature, indeed, and deserve a little description. The amphitheater at Bellevue Hospital will hold over a thousand persons, and every Saturday at half-past one it is crowded to its full extent. The bill of fare has been advertised previously on the college bulletin-boards, and it always includes a capital operation, often two or three of them. At the appointed hour Prof. Wood walks in. He is a little man with a very erect gait, a ruddy complexion, gray hair and moustache, and keen dark eyes. He dresses with the greatest care; his broadcloth coat, lavender trowsers, white vest and tie, and a button-hole bouquet are inseparable from his appearance in the amphitheater. "Jimmy" Wood, as he is endearingly called, is not a brilliant lecturer. His flow of words is interspersed with aphasic stops, and his voice is not a powerful one, still he gives a good many useful hints to the students, and no one can leave a course without feeling deeply the importance of preserving the periosteum. But it is as an operator that the Professor shines, and justly so. There is no surgeon in the city who knows better what to do, or whose knife goes so unerringly to the right spot. He used to be one of the

"lightning operators," removing thighs, et cetera, in eleven seconds. He operates in this style now occasionally, just to show the "boys" how it is done. When Mr. Callender, the English surgeon, was here last winter, Dr. Wood invited him to amputate a thigh before the students at his clinic. Mr. Callender accepted the invitation, and removed the extremity neatly and well, but with some deliberation. It so happened that Prof. Wood had another thigh all ready for himself to amputate. The patient was wheeled in, and the American professor exclaiming, "Now, I'll show you how I do it," with two or three flourishes, had off the extremity—to the great admiration and applause of the class. It was an exhibition of "Jimmy" Wood's weakest point, his vanity, as well as his surgical skill. This vanity is quite insatiable, but is conceded by its possessor, and has its amusing side. "Jimmy" is very popular with the young men of the profession. These he is always willing to help; in fact there is no surgeon in the city who has aided so many. In return, he exacts of his protegés implicit deference to himself and a sufficient amount of complimentary attention to feed the undisguised vanity of his nature. Dr. Wood is very successful in his surgical operations. Shrewdness and good judgment, a skillful knife and a scrupulous attention to after-treatment will explain this. He does not use the Lister method. His amputations are all treated with open dressings; and he has had such uniform and remarkable success that it would be absurd indeed to make a change. I cannot give the exact statistics, but I am told that he has had only one death from amputations in four or five years. He operates twenty or thirty times during the year. It should be remembered, too, that some of his cases have been treated in wards that used to be rank with puerperal fever.

The question of the dressing of wounds brings up that of Listerism. And it is a somewhat curious fact that while in London Lister is just receiving his greatest triumphs, in New York the method of Lister, pure and simple, is certainly dying out. At the recent discussion on Listerism, in London, only Bryant, Savory and Holmes opposed Lister and his spray. In this city the method is used in New York and Roosevelt Hospitals only by one surgeon. It is but little used in Bellevue, Presbyterian,

St. Luke's and St. Francis, Hospitals. At the Woman's Hospital, it is used as a rule in ovariotomy, but not always. Antiseptic surgery, that is to say, thorough cleansing and drainage are advocated, but Listerism, with its accompanying spray, is not considered essential nor always useful.

At the New York Hospital, a somewhat new and apparently very admirable method of treating compound fractures, has been introduced by Dr. Markoe. Putting them up in Lister was tried for some time, and it was thought at first to be attended with excellent results. But a wider experience shows the contrary. A compound fracture put up in Lister, has to be dressed perhaps a dozen times during the first week. Every time the dressing is renewed, the leg has to be disturbed, the fragments are moved and the parts irritated. The consequences are not good. The new method now adopted is to make a counter-opening to the wound in the injured limb, and to pass a large drainage-tube through this opening. The limb is then put up in a plaster of Paris dressing in which fenestræ are cut. Carbolized water is then injected through the drainage-tube every two hours during the first day, and three times a day after this. Care has to be taken not to run the tube next an artery, as hæmorrhage may follow. With this dressing there is a very moderate fever, the thermometer does not often rise above 102° or 103°, and in some cases it does not reach 100°. Nearly two hundred cases have been tried by this method and, it is stated, there has been only one death, that being in a very bad case. The treatment has diminished the number of amputations performed at the hospital. It certainly is a very rational one and deserves further trial.

A case at Bellevue Hospital in which rhinoplasty was performed, has lately excited much interest among the profession and the public. A young man had lost his nose from a lupoid affection. The progress of the disease had ceased, but the contractions of the scar had produced ectropion of both lids. This was relieved by plastic operations. An attempt was then made to restore the nose, the middle finger of the left hand being used as the agent. The matrix of the nail was first destroyed by caustic as a preliminary measure; an incision was then made along the median line of the palmar surface of the finger, flaps were dissected back and their edges stitched to the denuded sides of the nasal orifice. During the operation so much blood got into the larynx that tracheotomy had to be performed. After the operation, the finger was held in place by several straps of strong adhesive plaster. It is intended, after the adhesion has taken place, to ligate the digital arteries one after another. The finger will then be amputated, and the new nose perhaps further trimmed into good proportions. At last accounts the finger had united well in all parts except at the top. The deficiency here can be easily remedied. It is not to be expected that the boy will have an organ of Grecian beauty, but all who have ever seen the noses constructed from flaps alone will appreciate the advantage of having something solid and substantial, like a finger, upon which to work.

There is another similar case of nasal difficulty in the hospital, and if the present operations are successful, they will be repeated on the second patient. This particular rhino-plastic operation has been done once before in England, but never with the completeness of detail or in so bad a case as the present one. Dr. Sabine, the surgeon will gain much credit for originality and skill, if he succeeds.

A plastic operation has recently been performed at the New York Eye and Ear Infirmary which is quite novel and interesting. A boy about twelve years old had had his face greatly disfigured by a scar. One of his eyelids was almost entirely gone, the row of lashes being drawn up until nearly where the eyebrows had been before they were burnt off. Dr. Norris, the visiting surgeon, performed the following operation. He dissected off the skin from a large semilunar space just above the eyelashes of the injured side. He then dissected up a piece of skin from the side of the chest. It was semilunar in shape and about three inches long by two in width. This was dissected entirely off and laid carefully upon the denuded surface where the eyelid should be. It was held in place and the eye kept shut by layers of collodion painted over it. The whole piece united to the surface below it, all except the center, doing so by first intention. I saw the boy a couple of months after the operation. The large flap had then contracted down so that it was not more than an inch long by half an inch in width. The boy had an excellent eyelid, with very little disfigurement. The operation was certainly a great boon to the boy and is extremely creditable to the surgeon.

I was told by the house-surgeon, Dr. Oppenheimer, that benzoic acid which has recently been recommended so highly in eye diseases, deserves much of the credit which it is getting. It is particularly serviceable in corneal affections.

We have been having times of peace and love at the New York Academy of Medicine. A little while ago Mrs. Wm. Astor presented to the Academy, through its president, Fordyce Barker, a beautiful silver cup, to be used on great occasions and called "The Loving Cup." Its presentation was accompanied by a number of speeches. It cannot be said they were very eloquent or of high literary merit, but they gave expression to cordial feelings, and showed an appreciation of the fact that the medical profession is beginning to be estimated at a juster value by the intelligent among the laity. It is a fact, in cities at least, that doctors are both praised and blamed much more than they should be. The profession deserves a different judgment from that given it a generation ago. There are more men in it now of the best education, of fine scientific attainments and general culture. In spite of charlatanry outside and jealousy within, the character and standing of the average physician is higher. It is gratifying, therefore, to see evidences that this fact is getting to be understood.

A great deal has been said and written and done here, about lunacy reform. The medical profession is not, as a whole, very deeply aroused on the subject, although the *Medical Record* has been hammering away for a long time, and the New York Neurological Society has taken the matter up with great vigor. An unsuccessful petition, praying for investigation, was gotten up last year by this society. The senate committee, before whom some of its members appeared and testified, made a report very unfavorable toward taking steps to reform. To this report the Neurological Society have issued a reply in which they show very clearly, and with some vituperative emphasis, that they were unjustly treated and misrepresented in every way. They also sustain most of the charges they made. A bill is now before the assembly providing for three additional members of the State board of charities, who shall act as commissioners in lunacy. The bill is moderate and sensible; it simply creates a commission raised above political and personal influences, to supervise the management of insane asylums.

As regards the merits of the question, there can be no doubt that we are sadly in need of some change. We have 2,700 lunatics for instance, crowded into two asylums on the Islands in East River. These are under the superintendence of one medical man, with an insufficient corps of assistants, and with no opportunities whatever for carrying out proper treatment for the patients. It is simply one vast boarding-house. There are similar deficiencies in the way of medical attendence, crowded accommodations, etc., in other asylums of the State and the rapid yearly increase of the insane makes the future look unpromising. The great trouble, however, lies in the fact that no radical improvements can be made without incurring much expense, and many think that we are doing well enough now, and that the system will gradually improve if left alone. This is very doubtful and the propriety of having an efficient lunacy board to point out the deficiencies and gradually introduce a better system of care and treatment can hardly be questioned.

The subject of the abuses of medical charity is likely to be agitated here again, this time with a view to introducing the provident-dispensary system as a measure of relief. New York suffers greatly from the crowds of paupers, or would-be paupers, that fasten upon it, and try to live off its charity. No class of persons suffers more perhaps than physicians, whom a great many people are accustomed to think educated and established in order to furnish gratuitous medical relief. The story may have reached you of one of this class, who called in a carriage at a dispensary, and complained at having to wait for treatment, because she was *paying for the carriage by the hour.*

Provident-dispensaries are simply mutual health associations, whose members earn a weekly salary not exceeding ten dollars. They pay a weekly sum of four or five cents into a general fund, and are then supplied when sick, with medical attendance and nursing. Such institutions have been working for some time in many parts of England, and when they do not have to compete with free dispensaries that furnish clinical material for the medical colleges, they easily become self-supporting and successful. The medical men attending them get paid respectable sums, varying from $300 to $1000 per year. There are of course dangers and difficulties attending them, but there is no reason why they should not be successful and useful in most cases.

Our State is likely to have, at last, a State board of health. This we have needed for a long time. The difficulty of harmonizing State and municipal authority, however, was felt to be great and has prevented any strong efforts from being made. In addition to this there have been very efficient health boards in our larger cities, so that the need of a State organization has not been so much felt. The present bill, however, has been drawn up under the supervision of the New York City Health Board and receives its approval. It makes the usual provisions for sanitary care, the collection of statistics and investigation of scientific subjects. It does not undertake to regulate medical practice or to deal with the quacks, as the Illinois board does. Although this would be desirable eventually, it would be too much for a new organization.

The profession in this city are beginning to prepare (by subscription) for the annual meeting of the American Medical Association in June. Everything is being arranged for the expedition of business and the comfort and enjoyment of the guests. It is likely that the meeting will be a great success and a very enjoyable occasion.

New York has just closed one of its gayest seasons. This may appear to be a fact hardly worth recording in a medical journal, but it suggests a topic, the doctor in society, which might be made interesting. I don't know how it is in other large cities, but here the doctor is a very lively and ubiquitous element in every kind of social gathering. We have a large number of promising young men with "a small but growing practice" who have leisure on their hands, which they use for the delectation of the ladies. There isn't a club or reception, a sociable, a dinner, a dancing party, or a German, that hasn't its medical representative. This medical man-of-the-world pervades every variety of social gathering and ornaments all strata of society. He may be seen at the unassuming hops in a second-rate boarding-house, as well as at the dinner-tables of the wealthy and fashionable. And this is no exaggeration. There are some who are almost professional society men and "lady-killers." The majority, however, are of those who work hard enough during most of the time and like, once in a while, to look into the palpebral fissures of beauty in search of other things than morbid changes. Some are doubtless seeking, by what is called the "society dodge," to make themselves known. But it is a very uncertain method of gaining professional reputation, to be an expert waltzer. And whatever the motive or result, it is a fact that the young doctor is a brightly conspicuous figure in New York society. Some one has said that "the feeling which woman has for her physician is second only to that which she has for her lover;" and perhaps there is enough of truth in the saying to give the young medical man something of an advantage.

